# Risk factors for traumatic intracranial hemorrhage in mild traumatic brain injury patients at the emergency department: a systematic review and meta-analysis

**DOI:** 10.1186/s13049-024-01262-6

**Published:** 2024-09-17

**Authors:** Li Jin Yang, Philipp Lassarén, Filippo Londi, Leonardo Palazzo, Alexander Fletcher-Sandersjöö, Kristian Ängeby, Eric Peter Thelin, Rebecka Rubenson Wahlin

**Affiliations:** 1 Department of Emergency Medicine, Stockholm South General Hospital, Stockholm, Sweden; 2 Department of Clinical Science and Education Södersjukhuset, Karolinska Institutet, Stockholm, Sweden; 3 Department of Clinical Neuroscience, Karolinska Institutet, Stockholm, Sweden; 4 Department of Cardiac Surgery, Sant’Orsola-Malpighi Hospital, University of Bologna, Bologna, Italy; 5 Department of Neurology, San Raffaele Scientific Institute, Milan, Italy; 6 Department of Neurosurgery, Karolinska University Hospital, Stockholm, Sweden; 7 Medical Unit Neurology, Karolinska University Hospital, Stockholm, Sweden; 8 Department of Perioperative Medicine and Intensive Care, Karolinska University Hospital, Stockholm, Sweden

**Keywords:** Head trauma, Mild traumatic brain injury, Computed tomography, Traumatic intracranial hemorrhage

## Abstract

**Background:**

Mild traumatic brain injury (mTBI), i.e. a TBI with an admission Glasgow Coma Scale (GCS) of 13–15, is a common cause of emergency department visits. Only a small fraction of these patients will develop a traumatic intracranial hemorrhage (tICH) with an even smaller subgroup suffering from severe outcomes. Limitations in existing management guidelines lead to overuse of computed tomography (CT) for emergency department (ED) diagnosis of tICH which may result in patient harm and higher healthcare costs.

**Objective:**

To perform a systematic review and meta-analysis to characterize known and potential novel risk factors that impact the risk of tICH in patients with mTBI to provide a foundation for improving existing ED guidelines.

**Methods:**

The literature was searched using MEDLINE, EMBASE and Web of Science databases. Reference lists of major literature was cross-checked. The outcome variable was tICH on CT. Odds ratios (OR) were pooled for independent risk factors.

**Results:**

After completion of screening, 17 papers were selected for inclusion, with a pooled patient population of 26,040 where 2,054 cases of tICH were verified through CT (7.9%). Signs of a skull base fracture (OR 11.71, 95% CI 5.51–24.86), GCS < 15 (OR 4.69, 95% CI 2.76–7.98), loss of consciousness (OR 2.57, 95% CI 1.83–3.61), post-traumatic amnesia (OR 2.13, 95% CI 1.27–3.57), post-traumatic vomiting (OR 2.04, 95% CI 1.11–3.76), antiplatelet therapy (OR 1.54, 95% CI 1.10–2.15) and male sex (OR 1.28, 95% CI 1.11–1.49) were determined in the data synthesis to be statistically significant predictors of tICH.

**Conclusion:**

Our meta-analysis provides additional context to predictors associated with high and low risk for tICH in mTBI. In contrast to signs of a skull base fracture and reduction in GCS, some elements used in ED guidelines such as anticoagulant use, headache and intoxication were not predictive of tICH. Even though there were multiple sources of heterogeneity across studies, these findings suggest that there is potential for improvement over existing guidelines as well as a the need for better prospective trials with consideration for common data elements in this area.

*PROSPERO registration number* CRD42023392495.

**Supplementary Information:**

The online version contains supplementary material available at 10.1186/s13049-024-01262-6.

## Introduction

Traumatic brain injury (TBI) is an injury resulting from direct trauma or an acceleration-deceleration impact to the brain [1], with its most common causes being accidental falls, motor vehicle accidents, sports related accidents and violent crime [2]. It is a leading contributor to morbidity and mortality globally and ranks among the main causes of emergency department (ED) visits with over 60 million cases each year [3, 4].

An estimated 70–90% of TBI is mild traumatic brain injury (mTBI), defined as patients who present with an initial Glasgow Coma Scale (GCS) of 13–15 [3–5]. Previous studies have shown that around 10% of these patients will develop a traumatic intracranial hemorrhage (tICH) [6–9]. The presence of a tICH is associated with an increased risk of a deterioration requiring neurosurgical intervention [10, 11] and have also been shown to contribute to additional complications such as traumatic cerebral vasospasm [12]. Though several guidelines and management strategies exist for mTBI, there is significant variation in the risk factors accounted for in each guideline [8, 13–15]. Furthermore, there are previously reported issues with computed tomography (CT) overuse through application of existing guidelines which leads to a risk of unnecessary patient harm through radiation exposure [16], extended ED waiting times, higher healthcare costs [17–19], as well as an environmental burden in terms of carbon dioxide emissions [20]. Also, since the initial implementation of some of these guidelines, there has been changes in the prevalence of some existing risk factors such as the introduction of direct oral anticoagulants (DOACs) [21], and changing demographic trends such as an increasing population of elderly patients suffering from mTBI [4, 22]. In addition, as an alternative to current guidelines, several ongoing and completed studies look towards individualized risk estimation in tICH using novel data-driven approaches [23–26]. These factors suggest that a renewed assessment of the panorama of predictors of tICH is necessary to improve upon existing guidelines for ED management of mTBI.

The aim of the current systematic review and meta-analysis was to assess data from previously published studies to determine the current state of evidence in risk factors for tICH in mTBI patients at the ED. Beyond determining which variables are significant risk factors, our meta-analysis also provides an opportunity for quantitative comparison between risk factors, a key aspect that is important in the assessment of risk in individual tICH patients. Additionally, we sought to screen for novel risk factors that are not present in existing guidelines to provide a potential foundation for new variables to be considered in the development of new or revised management guidelines.

## Methods

This systematic review and meta-analysis was performed in accordance with the Meta-analysis of Observational Studies in Epidemiology (MOOSE) guidelines [27], and the Preferred Reporting Items for Systematic Reviews and Meta-analysis (PRISMA) guidelines [28]. The study was registered in the PROSPERO online database of systematic reviews [29] under the identification number CRD42023392495.

### Search strategy

MEDLINE, EMBASE and Web of Science were searched using variations of “mild traumatic brain injury”, “risk factor” and “traumatic intracranial hemorrhage” (complete search string and all variations are available in the supplementary material). The search was limited to publications from inception to June 6th, 2024. No geographical restrictions were applied to the search.

### Eligibility criteria

Inclusion criteria:

1. All retrospective, prospective, observational, and case–control studies reporting predictive variables in mTBI patients at the ED for head CT-verified tICH (traumatic epidural hemorrhage, traumatic subdural hemorrhage, traumatic subarachnoid hemorrhage, or traumatic intraparenchymal hemorrhage).
2. Patient populations were limited to 16 years and older to exclude patients that fall under pediatric TBI management guidelines.
3. The patient population in included studies were defined as mTBI with Glasgow Coma Scale (GCS) > 12 or where a subset of patients with GCS > 12 could be extracted from the presented data.
4. The minimum number of patients in each individual study required for inclusion was set to 50 in order to acquire an adequate number of observations per risk factor (based on the threshold chosen in previous literature [15]).
5. Only English language publications were included.

Exclusion criteria:

1. In order to avoid study populations with significantly skewed risk profiles in comparison to a general mTBI population, studies that only examined a subset of mTBI patients (for example only elderly patients, only patients on anticoagulants or antiplatelet medications) were excluded.
2. Review articles and articles with duplicate data were excluded.
3. Grey literature such as conference abstracts and unpublished data were excluded in favor of inclusion of only peer reviewed publications of sufficient quality.

### Data extraction

All titles, abstracts and full texts were screened by L.Y. and one other co-author (E.P.T., P.L., F.L. or L.P.) independently. Two independent assessors (L.Y. and P.L.) analyzed all full-text papers for suitable data to be included in the meta-analysis. Where results differed between the assessors a senior member of the review team was consulted to assist in reaching a consensus.

### Statistical analysis

Data on the impact of risk factors on tICH were extracted from the studies included and synthesized. The odds ratios (OR) were calculated using the Mantel–Haenszel method and the random effects model was used to calculate the pooled OR and 95% confidence intervals (CI) of the correlation of risk factors to tICH. Statistical heterogeneity was assessed using the I^2^ statistic. Funnel plots were produced to assess publication bias. All statistical analyses were performed in R (version 4.0.4, R Core Team, 2023).

### Risk of bias assessment

Two authors (L.Y. and P.L.) independently assessed the methodological quality of the included studies using the Newcastle–Ottawa Scale (NOS) [30]. Any disagreement was resolved through discussion to reach a consensus. The NOS examines three aspects of each study for a maximum score of nine stars. Studies were deemed to have a low risk of bias at nine stars, moderate risk of bias at seven to eight stars and high risk of bias at below seven stars.

## Results

### Study selection

**Fig. 1:**
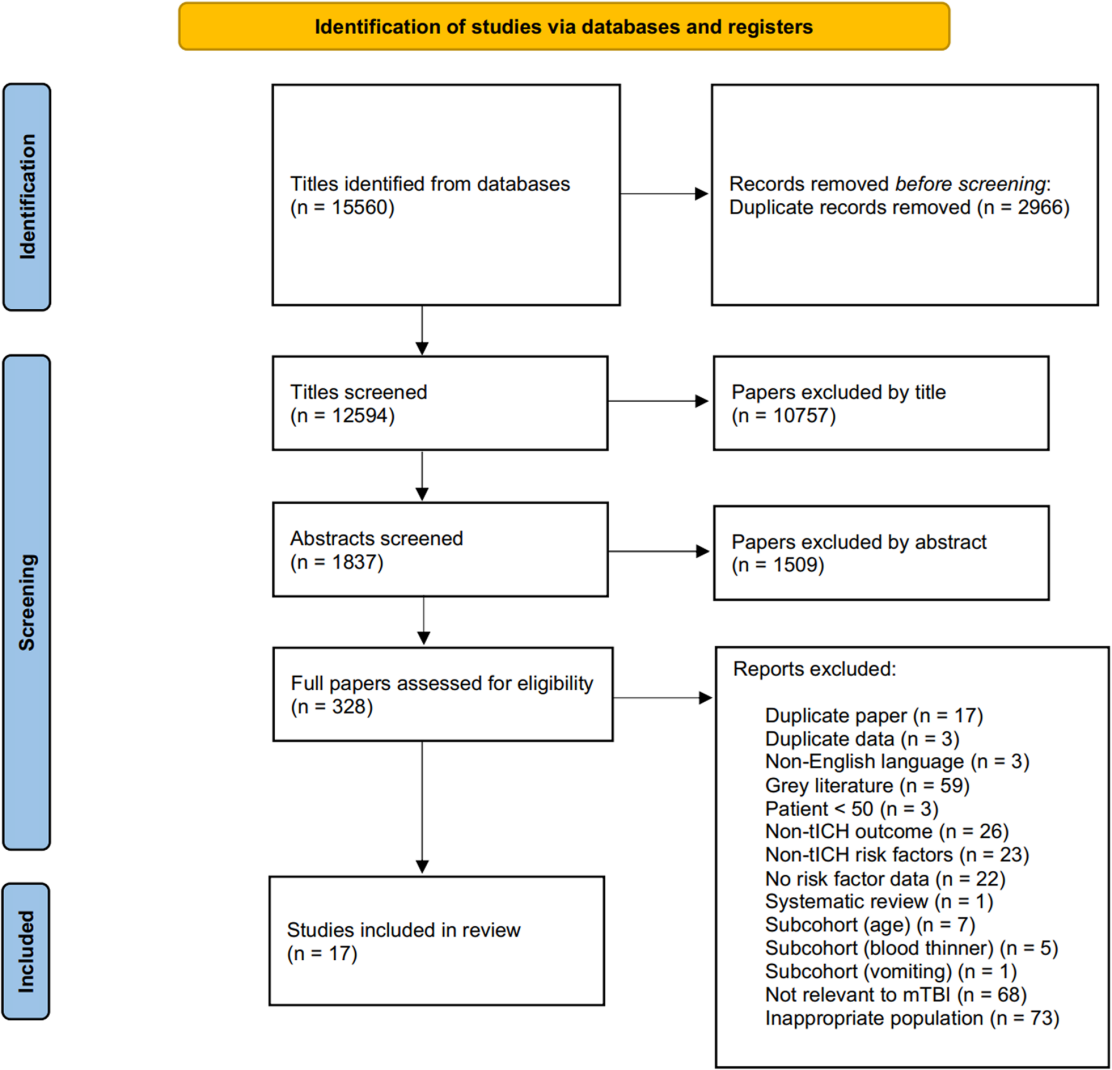
Preferred Reporting Items for Systematic Reviews and Meta-Analyses (PRISMA) flow chart of the study selection process for risk factors for traumatic intracranial hemorrhage. Out of total of 15,560 potentially relevant studies screened, a final total of 17 studies were included

The literature search yielded 15,560 titles, which after duplicate removal and title and abstract screening were narrowed down to 328 full papers of which 17 were included in the final review. The selection process in its entirety is shown in Fig. 1.

### Study characteristics and outcome

Out of the included studies, 6 were prospective and 11 were retrospective. All papers were published between 2011 and 2024. The studies were conducted in North America, Europe and Asia. The included publications contained a total of 26,040 mTBI patients that had undergone a head CT scan. The total number of patients with a CT-verified tICH was 2,054, resulting in a total tICH prevalence of 7.9% (Table 1).

**Table 1:** The characteristics of the selected studies

Author	Year	Country	Single/multi-center	Study design	Mild TBI definition	Primary outcome	Inclusion criteria	Exclusion criteria	Recruitment period	Mean age	Median age	Male percentage	tICH	Population	tICH prevalence
Bonney [31]	2020	United States	Single	Retrospective	GCS 13–15, with or without LOC or PTA	Intracranial hemorrhage including subacute hemorrhages. Isolated skull fractures excluded	Blunt head trauma, unspecified time interval after trauma, age 18 + , undergone head CT	Penetrating trauma	2010–2011	–	45 (30–58)	72.0%	477	5634	8.5%
Chayoua [32]	2024	The Netherlands	Multicenter	Prospective	GCS 13–15, maximum LOC 30 min and/or PTA 24 h	Intracranial hemorrhage corresponding to Marshall score > 1	Age 18 + . Selected for head CT based on CHIP rule	Significant neurologic or psychiatric comorbidity. Prior hospital admission for TBI. Drug abuse. Mental disability. Language barrier	2020–2022	–	48 (18–92)	57.7%	59	253	23.3%
Claudia [33]	2011	Italy	Single	Retrospective	GCS 14–15, normal neurological findings	Acute intracranial hemorrhage	Adult patients. Undergone CT	No CT performed	2007–2008	57 ± 25	–	52.7%	89	1410	6.3%
Galliazzo [34]	2019	Italy	Single	Retrospective	GCS 13–15	Acute intracranial hemorrhage	Age 18 +	On LMWH	2015–2017	–	71 (46–83)	50.1%	68	1846	3.7%
Haddadi [35]	2022	Iran	Single	Prospective	GCS 13–15, one or more of LOC max 30 min, PTA max 24 h, focal neurologic signs, nausea or vomiting	Acute intracranial hemorrhage	Age 18 +	Pregnancy, spinal cord injury, a history of psychosis, neurological disorders or cancer	2018–2019	–	–	–	30	89	33.7%
Hosseininejad [36]	2023	Iran	Single	Prospective	GCS 14–15	Acute intracranial lesion	Undergone head CT	Moderate and severe head trauma. Coagulation disorder. Surgical intervention	2018–2019	37 ± 4	–	58.1%	18	74	24.3%
Hsiao [37]	2017	Taiwan	Single	Prospective	GCS 14–15	Acute intracranial hemorrhage	Age > 16. Undergone head CT	Penetrating trauma. Age < 16. GCS < 13	2012–2013	58.1 ± 21.0	–	55.4%	154	1290	11.9%
Isokuortti [38]	2022	Finland	Single	Prospective	GCS 14–15	Hemorrhagic lesion on head CT	Age 18 + . Selected for head CT based on Scandinavian guidelines	–	2015–2016	–	70 (50–83)	47.0%	24	218	11.0%
Martinez-Rivas [39]	2023	Spain	Single	Retrospective	GCS 13–15	Acute intracranial hemorrhage	Undergone head CT	–	3 years (unspecified)	73.2 ± 19.0	–	49.8%	97	907	10.7%
Niklasson [40]	2024	Sweden	Multicenter	Retrospective	GCS 13–15	Acute intracranial hemorrhage	Head trauma, 18 +	Revisit, empty medical record, no physician participation, non-head trauma, multitrauma	2017, 2020–2021	–	70 (46–83)	50.8%	302	4850	6.2%
Nugraha [41]	2024	Indonesia	Single	Retrospective	GCS 13–15, maximum LOC 30 min and/or PTA 24 h	Acute intracranial hemorrhage	Age 18 + . CT performed	Associated injuries	2022–2023	–	33 (18–88)	63.4%	38	112	33.9%
Sakkas [42]	2023	Germany	Single	Retrospective	GCS 13–15, maximum LOC 30 min and/or PTA 24 h	Acute intracranial hemorrhage	Head trauma, craniofacial injury or cognitive alteration, adult	No cranial CT, GCS < 13, LOC > 30 min, PTA > 24 h, incomplete medical records	2016–2020	70.7 ± 21.1	–	55.3%	102	1837	5.6%
Savioli [43]	2020	Italy	Single	Retrospective	GCS 13–15	Acute intracranial hemorrhage	Age 18 +	ICH without head trauma, non-skull facial trauma, signs of skull fracture	2017–2018	64 ± 22.8	–	47.0%	195	2325	8.4%
Teeratakulpisarn [44]	2021	Thailand	Single	Retrospective	GCS 13–15	Acute intracranial hemorrhage	Age 18 + , head trauma and a risk factor	Other abnormal CT findings such as infarctions	2018	–	–	49.0%	24	100	24.0%
Uccella [45]	2020	Switzerland	Single	Retrospective	GCS 14–15	Acute intracranial hemorrhage	Chief complaint head injury	–	2014–2018	–	–	–	289	3358	8.6%
Vardar [46]	2022	United States	Single	Retrospective	GCS 14–15	Acute intracranial hemorrhage	Age 18 + , head CT performed	Transfer from outside facility, mechanism of trauma unknown, focal neurological finding	2020	80–2 ± 12.7	–	57.5%	63	1630	3.9%
Wolf [47]	2013	Austria	Single	Prospective	GCS 13–15	Acute intracranial hemorrhage	Blunt head trauma within 3 h from admission. Age 18 +	Penetrating head injury, unstable vital signs, focal neurological deficit, pregnancy, extracerebral injury, polytrauma, coagulopathy, cancer, multiple sclerosis	–	59 ± 23	–	56.1%	25	107	23.4%
Total													2054	26,040	7.9%

### Risk of bias

Using the NOS, 13 studies were found to have a high risk of bias while 4 studies were found to have a moderate risk of bias. Though many studies had appropriate selection processes and outcomes, there are significant issues in the comparability aspect of studies notably due to missing or insufficient confounding adjustment. The NOS scoring for each study with subcategory break-down is presented in Table 2.

**Table 2:** Risk of bias with the Newcastle–Ottawa assessment scale

Study	Selection	Comparability	Outcome	Risk of bias
Bonney 2020	***	–	***	High
Chayoua 2024	***	–	***	High
Claudia 2011	***	–	***	High
Galliazzo 2019	***	*	***	Moderate
Haddadi 2022	***	–	***	High
Hosseininejad 2023	***	–	***	High
Hsiao 2017	**	–	***	High
Isokuortti 2022	***	–	***	High
Martinez-Rivas 2023	**	–	***	High
Niklasson 2024	***	*	***	Moderate
Nugraha 2024	***	–	***	High
Sakkas 2023	****	*	***	Moderate
Savioli 2020	**	*	***	High
Teeratakulpisarn 2021	**	*	***	High
Uccella 2020	**	–	***	High
Vardar 2022	***	–	***	High
Wolf 2013	****	*	***	Moderate

The Newcastle Ottawa Scale is based on a number of stars assigned per category. * is one star, ** is two stars, *** is three stars, and - is no stars assigned

### Data synthesis of risk factors

**Fig. 2:**
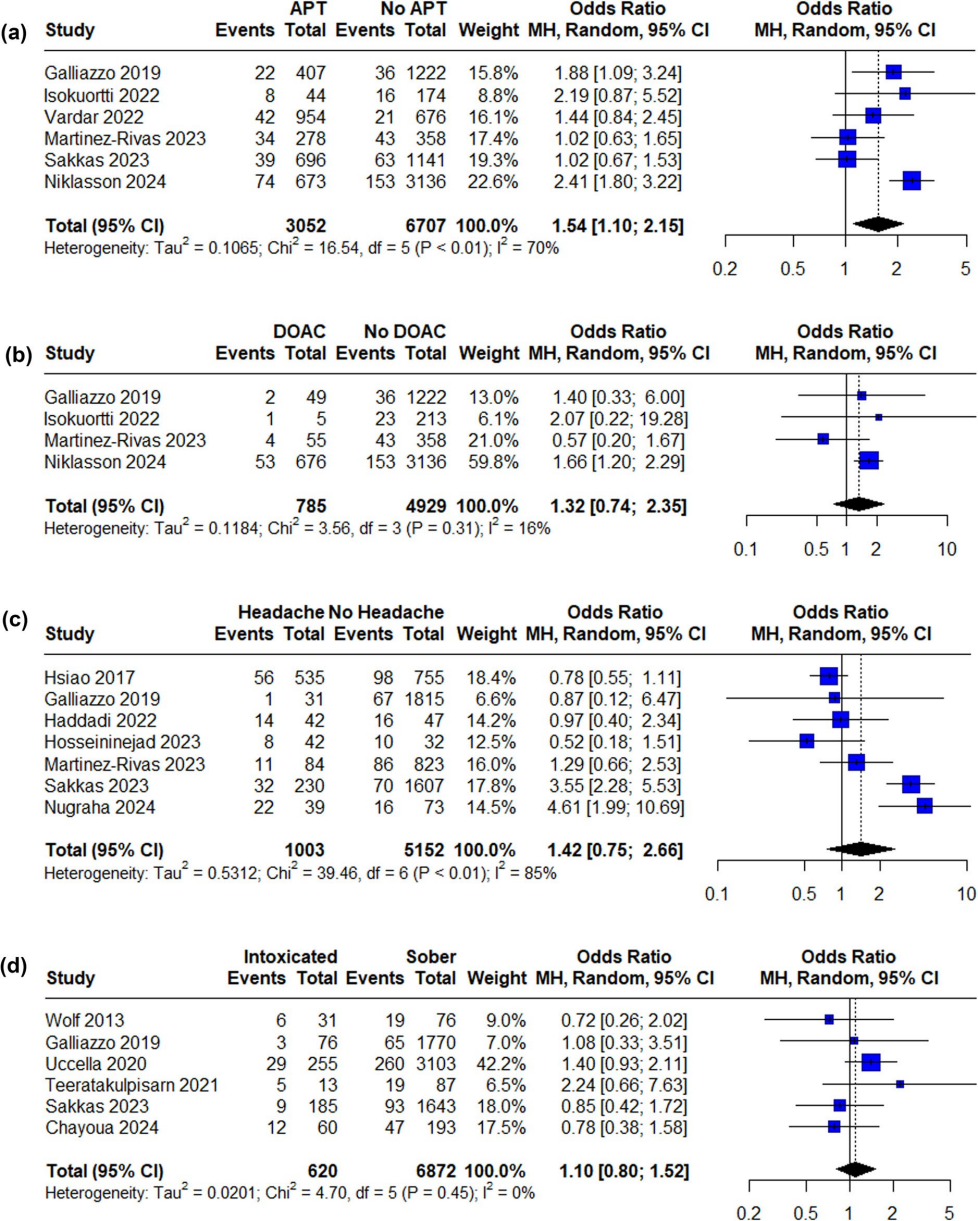

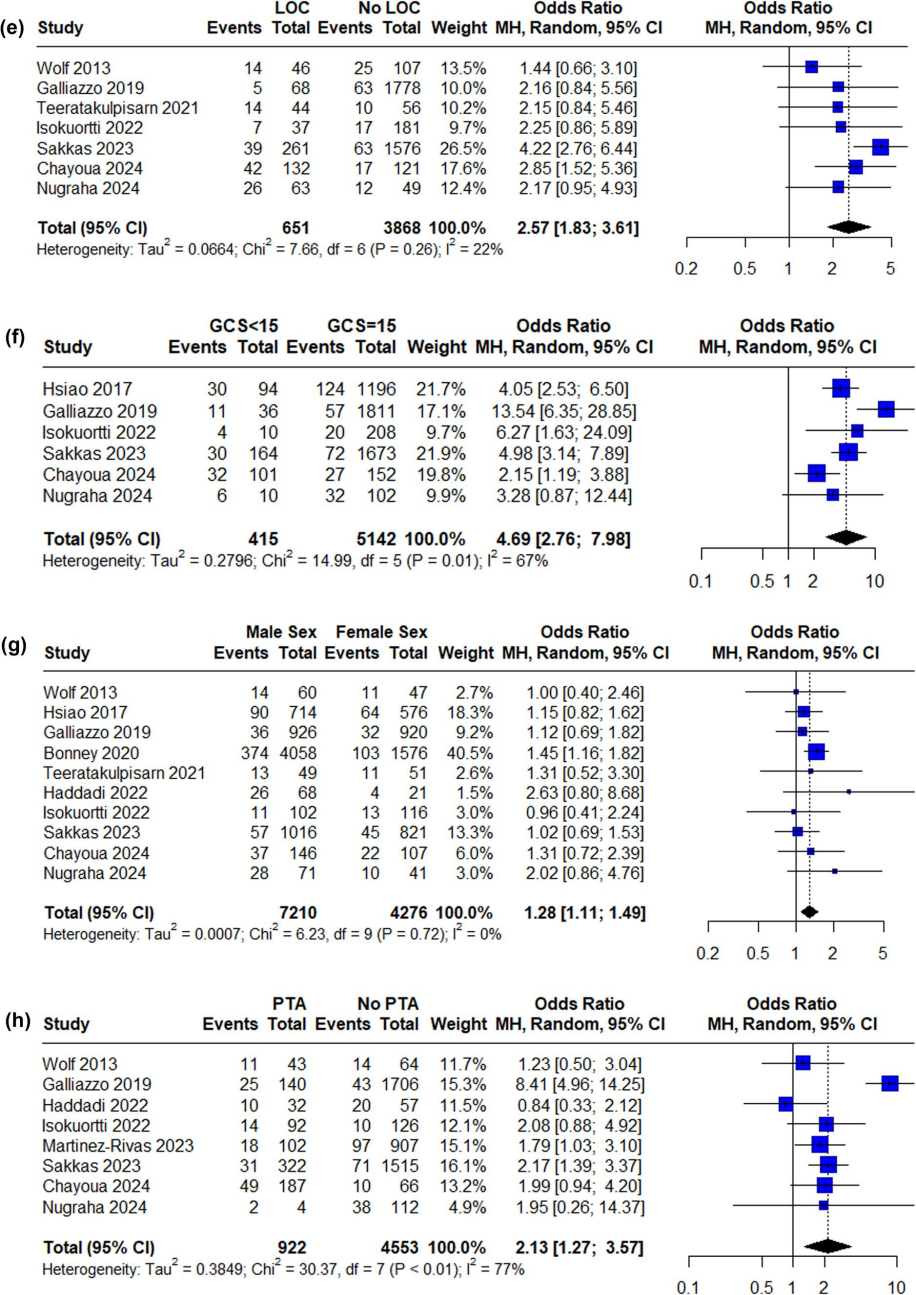

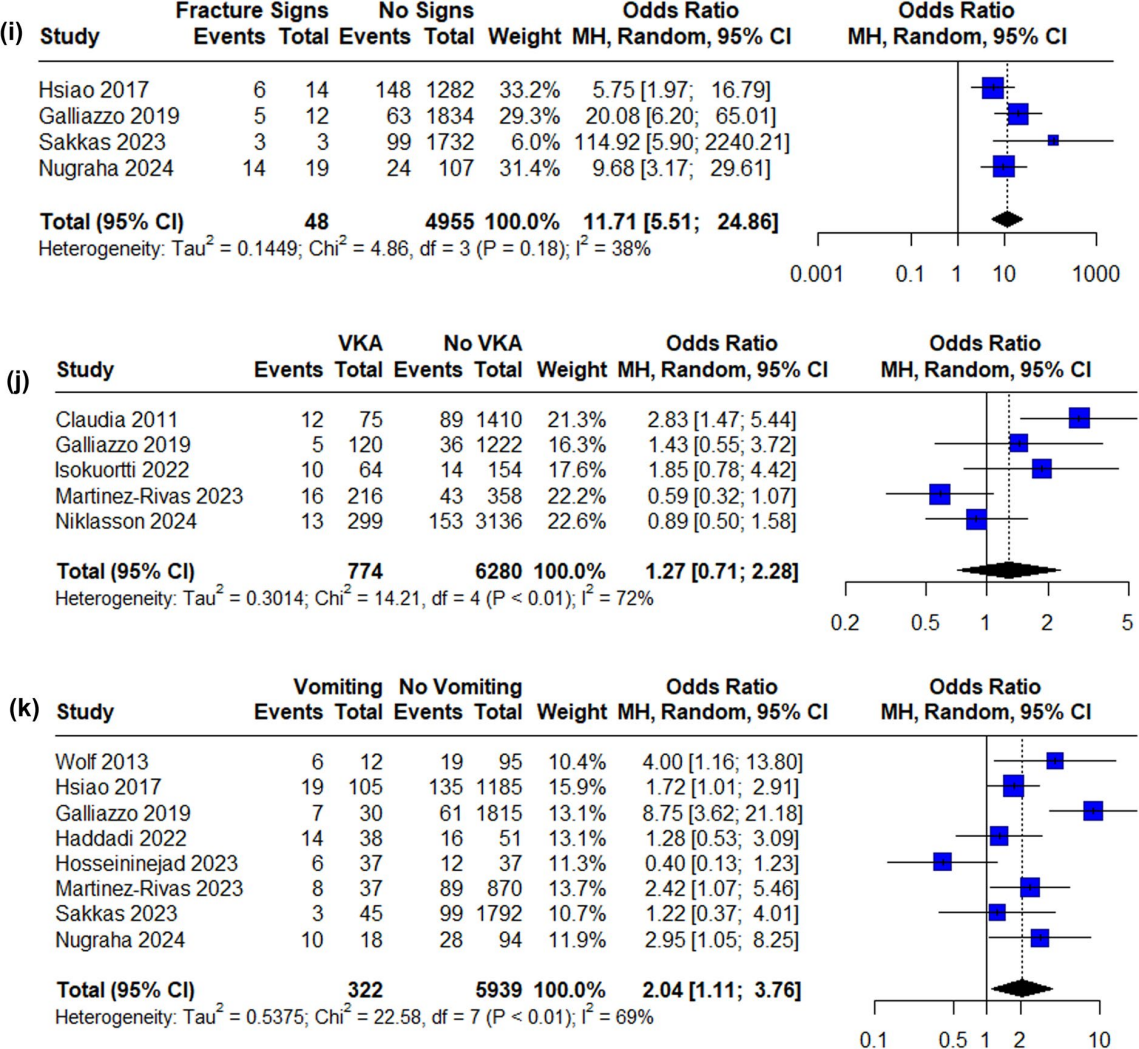
Forest plot of pooled ORs of tICH in patients **A** on antiplatelet medication, **B** on direct oral anticoagulation medication, **C** presenting with post-traumatic headache, **D** intoxicated, **E** loss of consciousness, **F** GCS below 15, **G** of male sex, **H** presenting with post-traumatic amnesia, **I** presenting with signs of skull fracture, **J** on vitamin K antagonist medication, **K** presenting with post-traumatic vomiting (OR, odds ratio, CI, confidence interval, MH, Mantel–Haenszel, APT, antiplatelet treatment, DOAC, direct oral anticoagulation, LOC, loss of consciousness, PTA, post-traumatic amnesia, VKA, vitamin K antagonist)

**Fig. 3:**
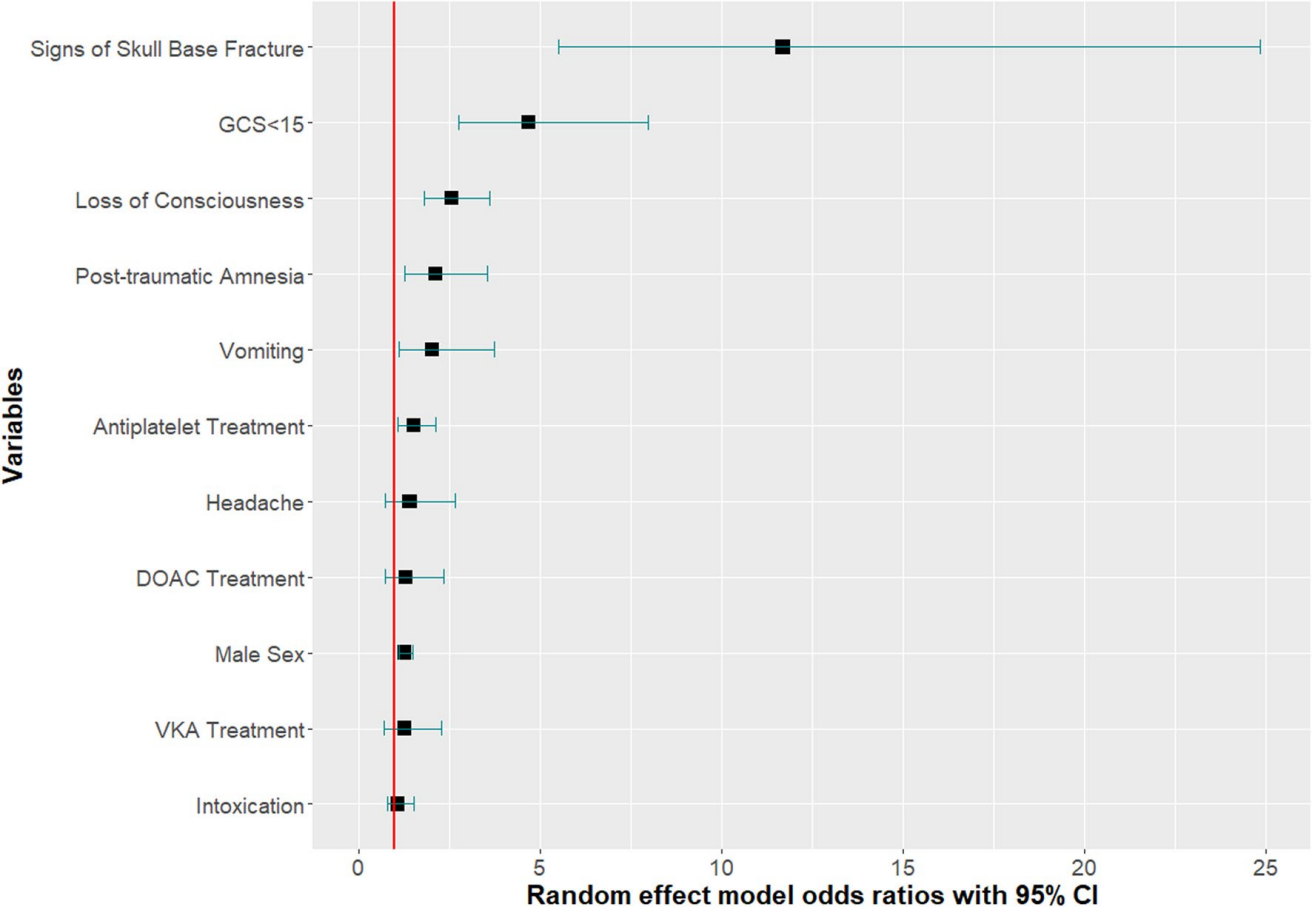
Forest plot of random effect model odds ratios with 95% CI of each risk factor from the meta-analysis (CI, confidence interval)

Eleven independent risk factors were available for data synthesis based on available data (Figs. 2, 3. The strongest predictor of tICH was signs of a skull base fracture (OR 11.71, 95% CI 5.51–24.86), followed by GCS < 15 (OR 4.69, 95% CI 2.76–7.98). Loss of consciousness (LOC), post-traumatic amnesia (PTA), vomiting, antiplatelet treatment and male sex were statistically significant risk factors for tICH in the meta-analysis. Funnel plots and Egger’s test were produced for each risk factor where possible and the results suggested no significant publication bias with the only exception of LOC (Egger’s test p = 0.0156). See supplementary material.

Several studies also assessed clinical variables currently not included in existing guidelines such as usage of serotonergic antidepressants [38], previous neurological and neurosurgical conditions (epilepsy, stroke, neurosurgery, cerebral neoplasia) [34], and multiple injuries [44], however, none of these were statistically significant predictors of tICH. Interestingly, the presence of a scalp lesion (defined as an ecchymosis or hematoma over the scalp) as a clinical finding was identified in one study as a significant predictor of tICH (OR 2.2, 95% CI 1.03–4.68) [34].

## Discussion

This systematic review and meta-analysis examined predictive risk factors for tICH to characterize their roles in guidelines for the acute stage management of mTBI. A total of 17 studies spanning global research sites in North America, Europe and Asia were assessed for available data. No studies representing Africa, South America or Australasia were included in the final list of studies for review. Out of the eleven independent risk factors available for data synthesis, seven were shown in the meta-analysis to have predictive value for tICH and a few novel variables were shown to be statistically significant in individual studies. We believe that these findings may prove useful in the validation of existing guideline elements as well as provide justification for data elements in future studies. The risk factors we were able to provide a meta-analysis for are discussed in more detail below.

### Signs of a skull base fracture

The strongest risk factor for tICH in our meta-analysis was signs of a skull base fracture, a known significant clinical sign present in the Canadian CT Head Rule [8], New Orleans CT Head Trauma Rule [14], CT in Head Injury Patients Rule [7], and the Scandinavian Neurotrauma Committee Guidelines [15]. The relative strength of this risk factor in our data must be interpreted with caution, as its low incidence leads to a wide confidence interval. Scandinavian guidelines does take the relative specificity of this predictor into account however, by including it among higher risk predictors where a 24 h minimum admission for in-hospital observation is recommended regardless of CT findings [15].

### Decreased GCS

A post-traumatic decrease in GCS was a significant risk factor for tICH among the variables in our meta-analysis. Most existing TBI guidelines account for GCS reduction in some manner [8, 14, 15, 48], with some variations in recommendations ranging from Haydel et al*.* in the New Orleans CT Head Trauma Rule recommending all patients with GCS < 15 undergoing a CT [14] to Undén et al*.* in the Scandinavian Neurotrauma Committee’s guidelines suggesting a minimum of S100B sampling or 12 h of observation as possible alternatives to a head CT in low risk GCS 14 patients [15].

### Loss of consciousness, post-traumatic amnesia and vomiting

LOC and PTA are both criteria in some management algorithms and a prerequisite for some definitions of mTBI [3, 8, 14, 49], and both were statistically significant predictors in our meta-analysis. There is some variation in the view on these predictors across different regions, such as in the Scandinavian Neurotrauma Committee’s guidelines for management of TBI which includes LOC and recurrent vomiting as indications for CT or S100B sampling, but not PTA [15]. There are some pragmatical difficulties in the accurate assessment of amnesia as a clinal variable as it may include any combination of transient, anterograde, retrograde amnesia, as well as coinciding with loss of consciousness. Foks et al*.* have shown in a head injury population with only GCS 15 patients with and without having undergone a head CT that PTA is associated with traumatic findings on CT with an OR of 3.8 (95% CI 2.9–4.9) and when combined with loss of consciousness the OR increases further to 4.1 (95% CI 3.1–5.3) [50]. Smits et al. have in a cohort of patients with GCS 15 and one additional risk factor shown that anterograde amnesia is not associated with hemorrhage, but persistent retrograde amnesia does show an association at OR 1.7 per 60 min of time with amnesia [51]. As expected, vomiting was a significant predictor of tICH in our meta-analysis. This is consistent with it being a known risk factor for tICH and is also included in existing management algorithms for tICH [8, 14, 15, 48].

### Antiplatelet treatment and anticoagulation

Interestingly, though antiplatelet treatment was a significant risk factor in our meta-analysis, VKA and DOAC treatment were not. Existing guidelines caution careful management of patients on all categories of medication that impact hemostasis [8, 14, 15], and a number of studies including two independent meta-analyses verify the significance of antiplatelet treatment in the context of hemorrhage risk [52–54]. Additionally, the finding that antiplatelet treatment seem to outweigh anticoagulation in terms of tICH risk has also been observed in multiple recently published studies [40, 48].

One aspect we hoped to be able to investigate in our meta-analysis was the impact of the shift in anticoagulation prescription from vitamin K antagonists (VKA) to DOACs. The results from our meta-analysis suggest that VKA and DOAC treatment were similar in their risk profile for tICH and were both non-significant (OR 1.27, 95% CI 0.71–2.28 and OR 1.32, 95% CI 0.74–2.35 respectively). Several studies contained data on DOAC treated mTBI patients during our screening process, but did not fit our selection criteria due to patient selection (anticoagulanted patient subcohorts [55, 56]). However, these studies do suggest a lower tICH risk in patients on DOAC in comparison to patients on VKAs. Also, in a study updating the CT in Head Injury Patients Rule based on multicenter patient data by van den Brand et al. [48], the patient data suggested anticoagulation after the introduction of DOAC was no longer a predictor of tICH. Similarly, a systematic review synthesizing data from anticoagulated mTBI subpopulations by Karamian et al. showed an overall incidence of tICH in mTBI patients on DOACs of 6.4%, lower than in mTBI patients on VKAs at 10.5% [57].

Considering the otherwise predominant consensus in several existing guidelines that anticoagulation contributes to increase risk of traumatic hemorrhage, one possible explanation for these findings is that there is a tendency among clinicians to order CT scans for all patients on anticoagulation, regardless of severity of injury and a lack of symptoms or other factors to justify a radiological examination. These findings warrant consideration in future guideline updates as well as studies.

### Headache and intoxication

The risk factors headache and intoxication are both included in the New Orleans Charity Head Trauma Rule [14], however both were not shown to be predictive of tICH in our meta-analysis. The inclusion of these variables is likely to be a factor in the consistent finding from previous validation studies that this guideline is low in sensitivity. This has been shown in pooled data in a systematic review and meta-analysis by Alzuhairy et al. to be 12.3% (95% CI 7.4–19.8%) [58]. Our results suggest that future guidelines that aim to reduce CT overuse in mTBI patients at the ED should prioritize other predictors than headache and intoxication, and these variables are not likely to be key candidates for standard data collection and confounding adjustment in future mTBI studies.

### Male sex

Male sex is the only risk factor detected in the meta-analysis to not be currently in use in one or more existing guidelines for management of mTBI. However, this finding is consistent with data previously published in Dunning et al. [59]. The percentage of male patients across studies included in this review ranged from 47 to 72%, suggesting differences in cohort compositions between studies and geographical regions. It is possible that these findings are secondary to the male population being subject to confounding from for example different trauma mechanism, however this is generally not explored in our included studies. Even though it is unlikely that these findings will directly impact clinical guidelines considering the impracticality of unselectively providing CT head scans to all male patients, male sex could be a variable up for consideration in a system akin to the application of female sex in the CHA_2_DS_2_-VASc score for atrial fibrillation [60].

### Age

Another risk factor we aimed to analyze in our meta-analysis was the aging population, and this has previously been partially accounted for in existing guidelines recommending thresholds of 60 [14] and 65 [8, 15] respectively. Two studies in our review reported data at the 65 year threshold (OR 1.14, 95% CI 0.66–1.96 [34] and OR 1.24, 95% CI 0.89–1.74 [37]), one study reported data at the 75 year threshold (OR 2.57, 95% CI 1.83–3.63 [43]), while an additional study reported data in terms of per year increase (OR 1.04, 95% CI 1.00–1.09 [47]). Surprisingly, Niklasson et al. found in their study population that an age threshold as low as 45 years of age proves to be a statistically significant predictor of tICH (adjusted OR 3.54, 95% CI 2.33–5.38) [40]. Though the variations in presentation made data synthesis unfeasible, the cumulative results suggest that though an older age appears to be a risk factor for tICH, there is insufficient data to support an optimal threshold for clinical application.

### Biomarkers

Though some biomarkers were assessed in the studies included in our meta-analysis, there was not sufficient homogeneity of method or data to perform data synthesis on individual biomarkers. Acute and non-acute stage biomarker use in mTBI has been characterized in other systematic reviews [61, 62], demonstrating significant potential. However Visser et al. suggest that there is even more variety in methodology in this subgroup of studies in terms of time points (relative to trauma and sampling), controls, cut-offs, and management of samples, which further generates difficulty in drawing robust conclusions [62].

### Scalp lesion

Galliazzo et al*.* reported the clinical finding of a scalp lesion as a statistically significant predictor of tICH [34]. In previous studies, the New Orleans CT Rule proposes that any sign of injury above the clavicle level as an indication for CT head scan in the GCS 15 mTBI population [14]. The association of scalp lesion with tICH suggests there may be additional approaches to risk assessment based on the external signs of injury using the localization of the injury that could be studied further.

### Limitations

This systematic review with a meta-analysis was conducted while balancing two major factors: heterogeneity and available data quantity. Heterogeneity poses a concern to the generalizability of our findings, and the following are several examples of this in our study material.

Firstly, the definition of mTBI is also known to be highly variable in literature [3], with both GCS 13–15 and 14–15 being frequently used in combination with other symptoms. Secondly, individual studies varied in their application of inclusion and exclusion criteria, ranging from specific comorbidities such as mental disability that hinder assessment [32] to missing or incomplete medical records [40, 42]. Thirdly, there is also significant variation in the incidence of hemorrhage (3.7–33.9%), as well as in the mean and median ages (37 to 80.2 and 33 to 77, respectively) in the study cohorts. We have presented these factors for each included study in Table 1 to clarify the differences between studies.

These causes of heterogeneity are known issues that have been reported in previous meta-analyses on mTBI [53, 57, 59, 61, 63, 64]. However, in our study we have applied a more stringent approach to study selection in comparison to these studies. Though it is impossible to eliminate all heterogeneity, we sought to minimize the impact of these issues on our findings by excluding studies that contain patient selection criteria that affect risk factors of interest. An example of this is the exclusion of articles that only include subcohorts of mTBI patients on anticoagulation [55, 56]. This is performed at the cost of reducing the number of studies that can be included in the meta-analysis, and careful consideration was made on an article-by-article basis to determine the benefit of inclusion versus exclusion.

For future studies on mTBI and tICH, we suggest application of standardized methods of collection of predictive variables and more homogenized structures of confounding adjustment based on existing guidelines and data on risk factors. This form of standardization would help to eliminate persisting issues with both comparability and generalizability across mTBI studies as well as in future literature reviews.

## Conclusion

The findings from this study provide additional context to risk factors currently in use as components in guidelines for the management of mTBI in the ED setting, contrasting high risk predictors such as signs of a skull base fracture with variables shown to have limited predictive capabilities for tICH such as anticoagulant use, post-traumatic headache and intoxication. Though these findings require further investigation, we have demonstrated that the methodology of a structured systematic review and meta-analysis could be applied to identify problematic aspects and serve as a foundation for updating existing guidelines.

## Supplementary Information

Additional file 1 1. Funnel plots and Egger’s tests. 2. Literature search strategy

## Data Availability

All data will be made available upon reasonable request.

## Abbreviations

TBI: Traumatic Brain Injury
mTBI: Mild Traumatic Brain Injury
tICH: Traumatic Intracranial Hemorrhage
LOC: Loss of Consciousness
PTA: Post-traumatic Amnesia
APT: Antiplatelet Treatment
DOAC: Direct Oral Anticoagulant
VKA: Vitamin K Antagonist
MOOSE: Meta-analysis of Observational Studies in Epidemiology
PRISMA: Preferred Reporting Items for Systematic Reviews and Meta-analysis
CT: Computed Tomography
GCS: Glasgow Coma Scale
CI: Confidence Interval
OR: Odds Ratio
NOS: Newcastle-Ottawa Scale
NSE: Neuron-Specific Enolase
