# Supplementary material for: Risk factors for traumatic intracranial hemorrhage in mild traumatic brain injury patients at the emergency department: a systematic review and meta-analysis
Source: Scand J Trauma Resusc Emerg Med. 2024 Sep 17;32:91. doi: 10.1186/s13049-024-01262-6 (PMC11406809; doi:10.1186/s13049-024-01262-6)
Supplement: Supplementary file 1 — Additional file 1 1. Funnel plots and Egger’s tests. 2. Literature search strategy [file 13049_2024_1262_MOESM1_ESM.docx]

**1. Funnel Plots and Egger’s Tests**

Male Sex


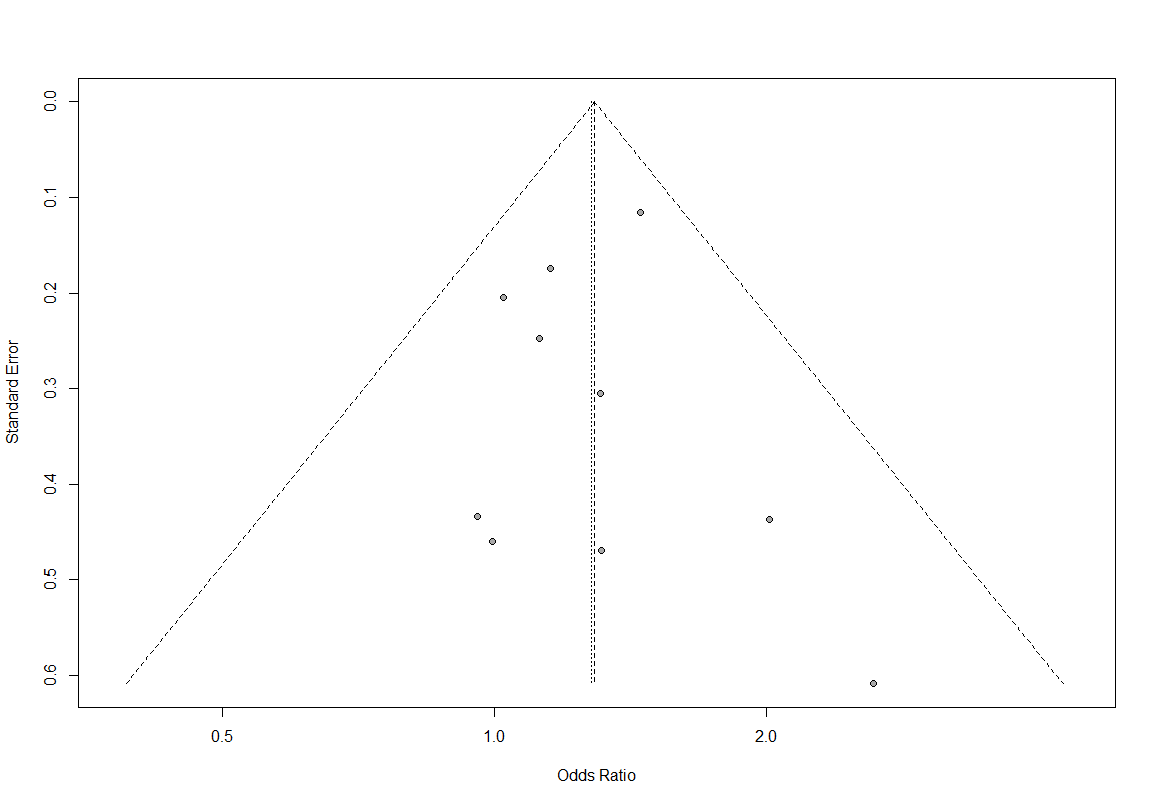


Egger’s test result: t = -0.07, df = 8, p-value = 0.9481

Bias estimate: -0.0383 (SE = 0.5700)

GCS


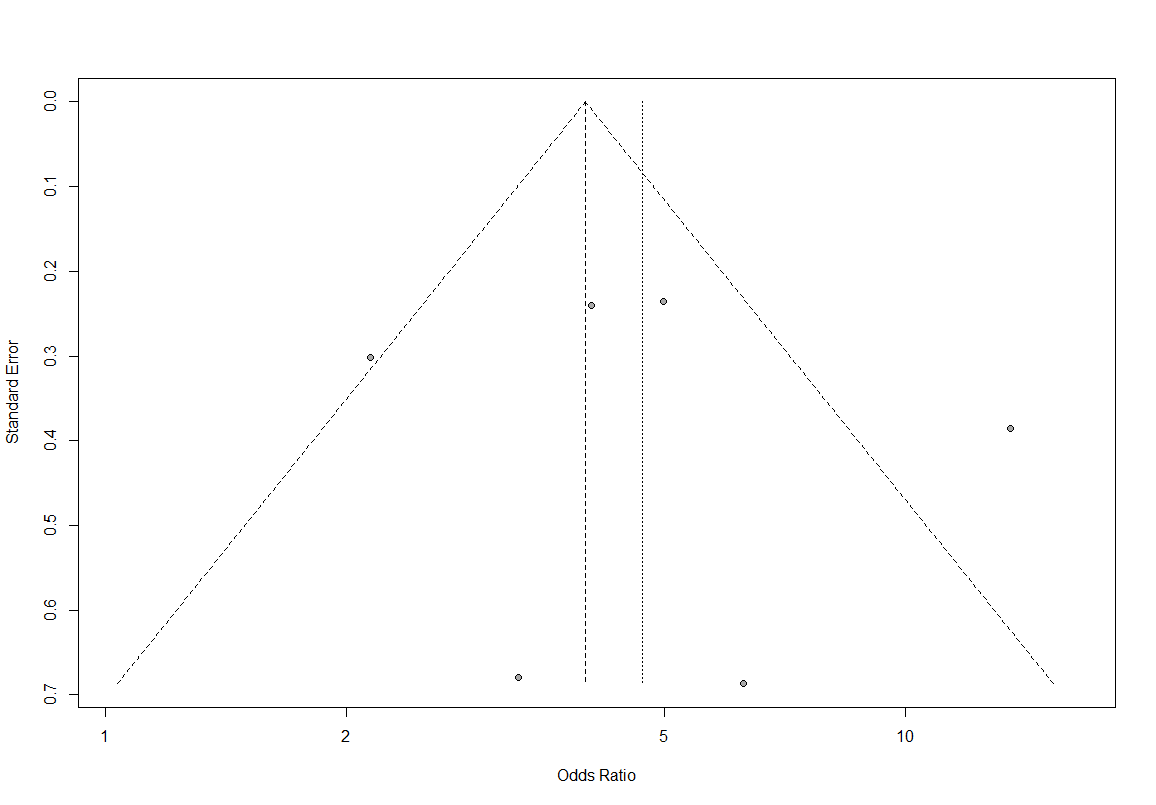


Egger’s test result: t = 0.36, df = 4, p-value = 0.7341

Bias estimate: 0.7681 (SE = 2.1085)

PTA


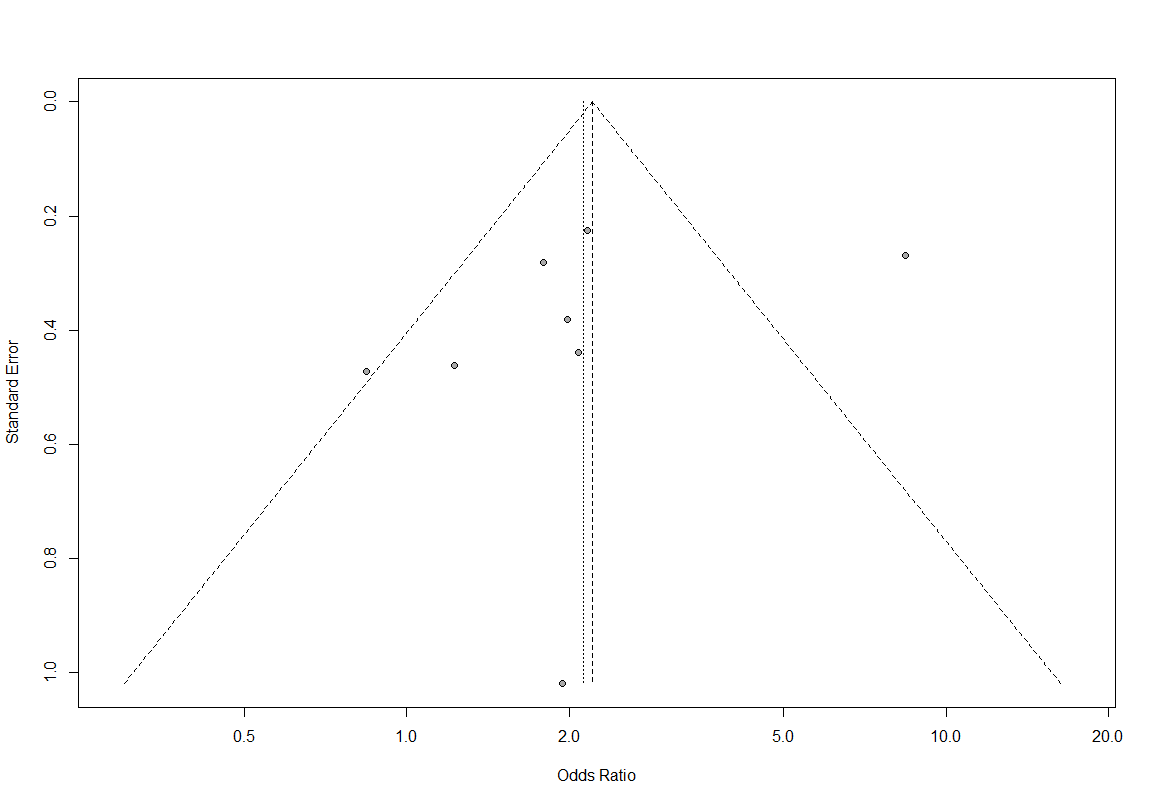


Egger’s test result: t = -0.96, df = 6, p-value = 0.3762

Bias estimate: -1.9996 (SE = 2.0926)

LOC


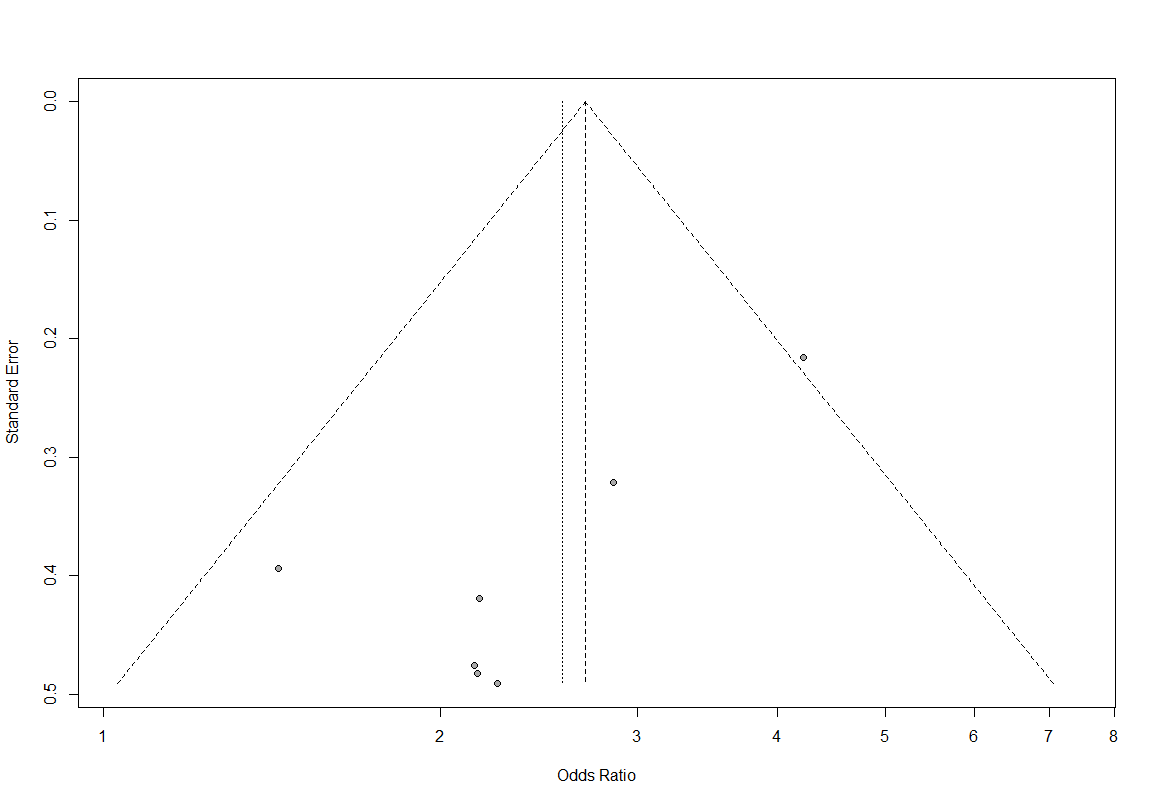


Egger’s test result: t = -3.59, df = 5, p-value = 0.0156

Bias estimate: -2.9176 (SE = 0.8116)

Intoxication


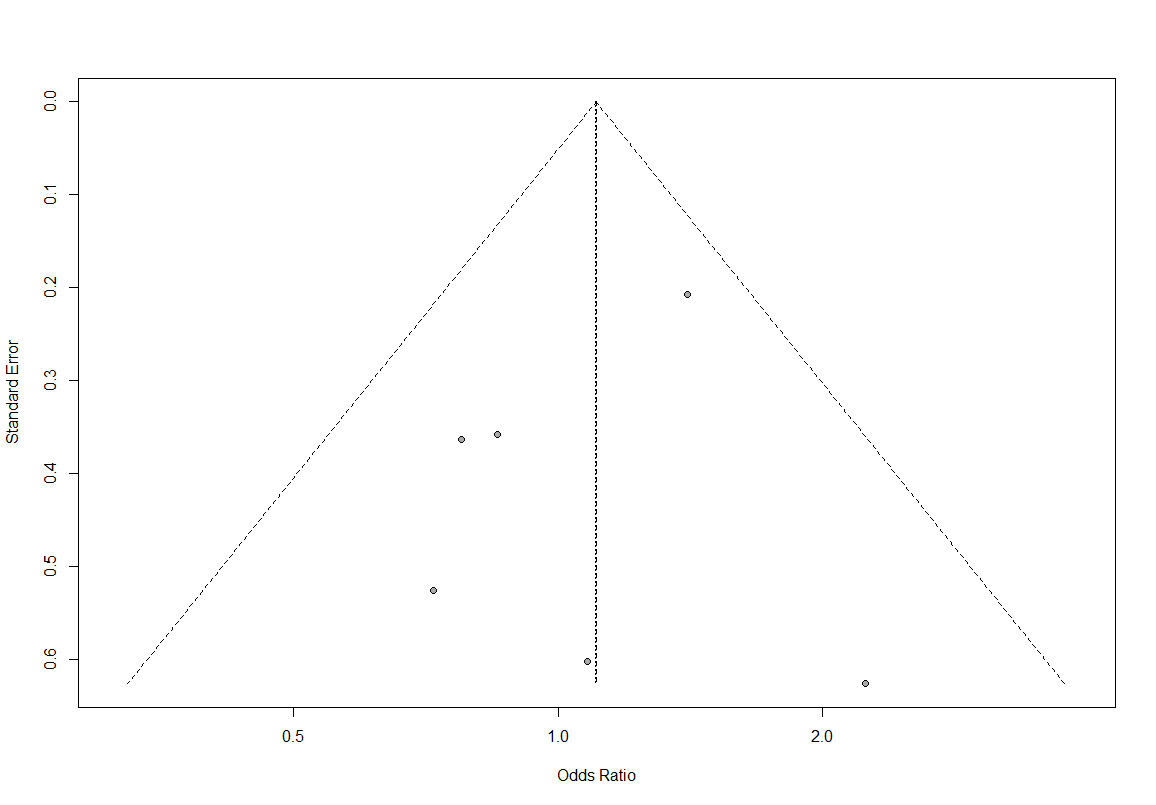


Egger’s test result: t = -0.61, df = 4, p-value = 0.5740

Bias estimate: -0.6575 (SE = 1.0754)

Signs of Skull Base Fracture


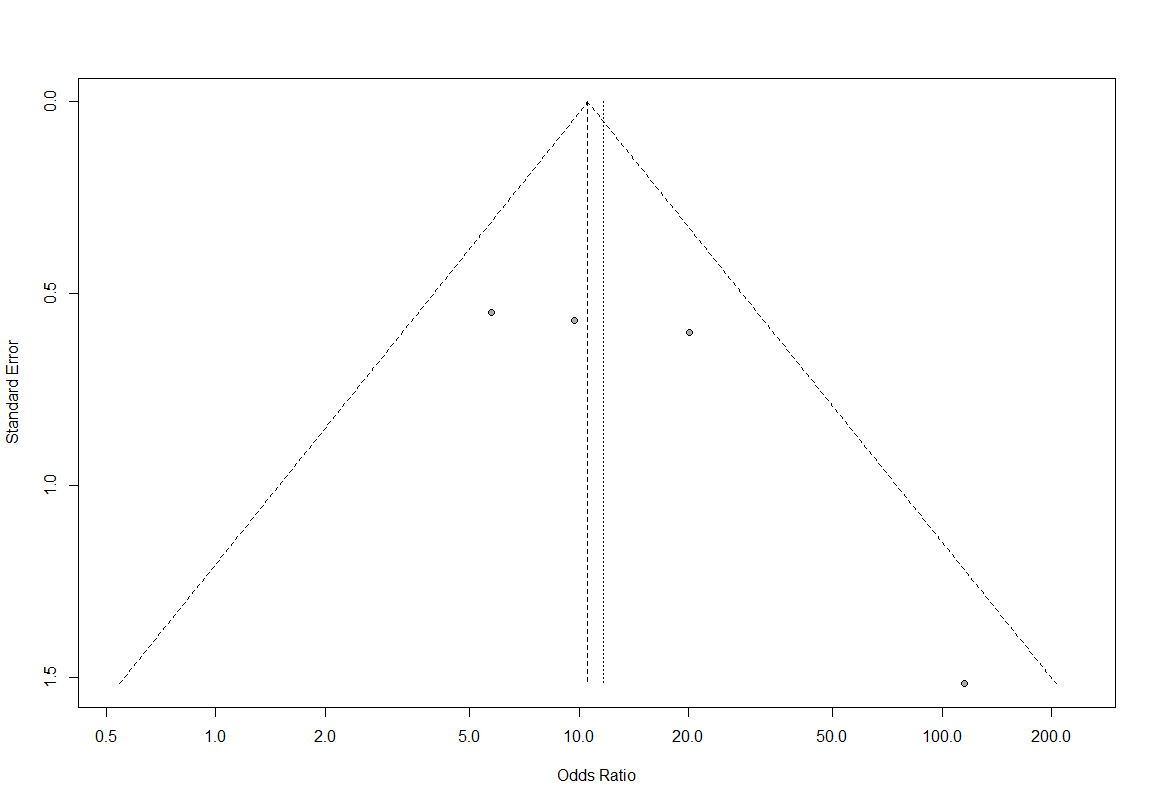


Too few studies for conducting an Egger’s test.

Vomiting


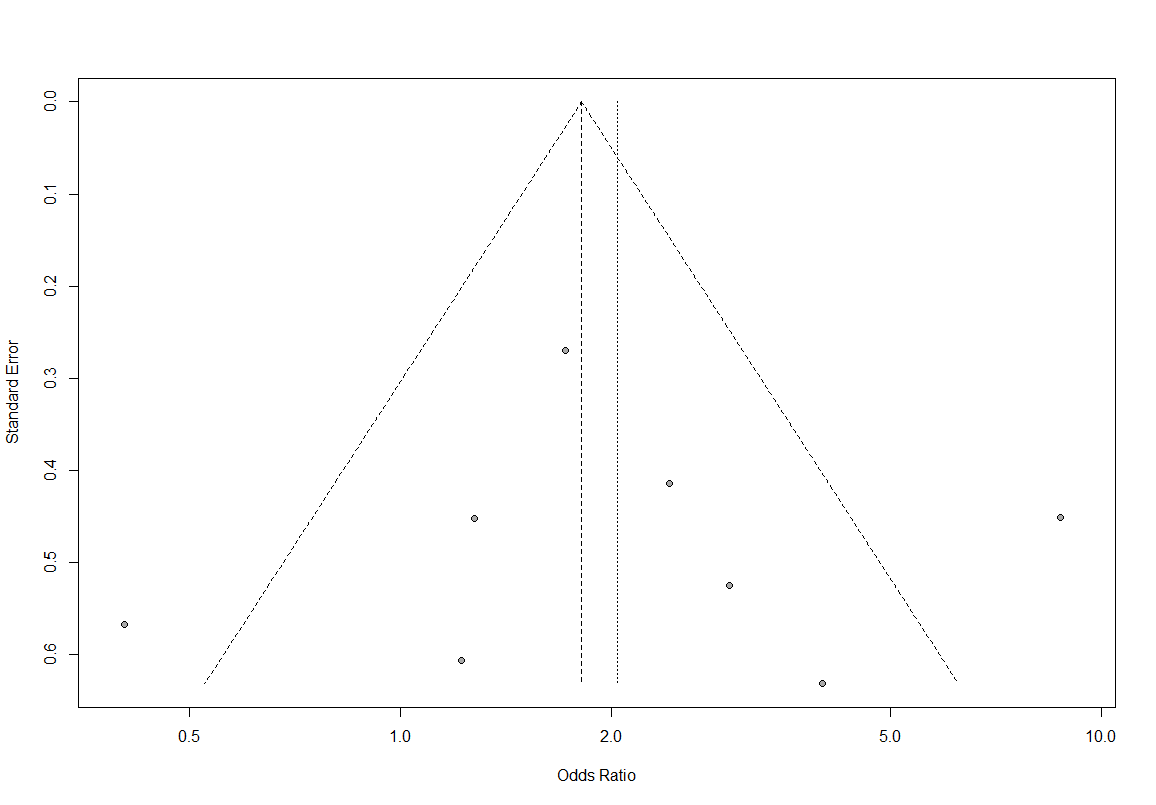


Egger’s test result: t = -0.00, df = 6, p-value = 0.9964

Bias estimate: -0.0113 (SE = 2.4319)

Headache


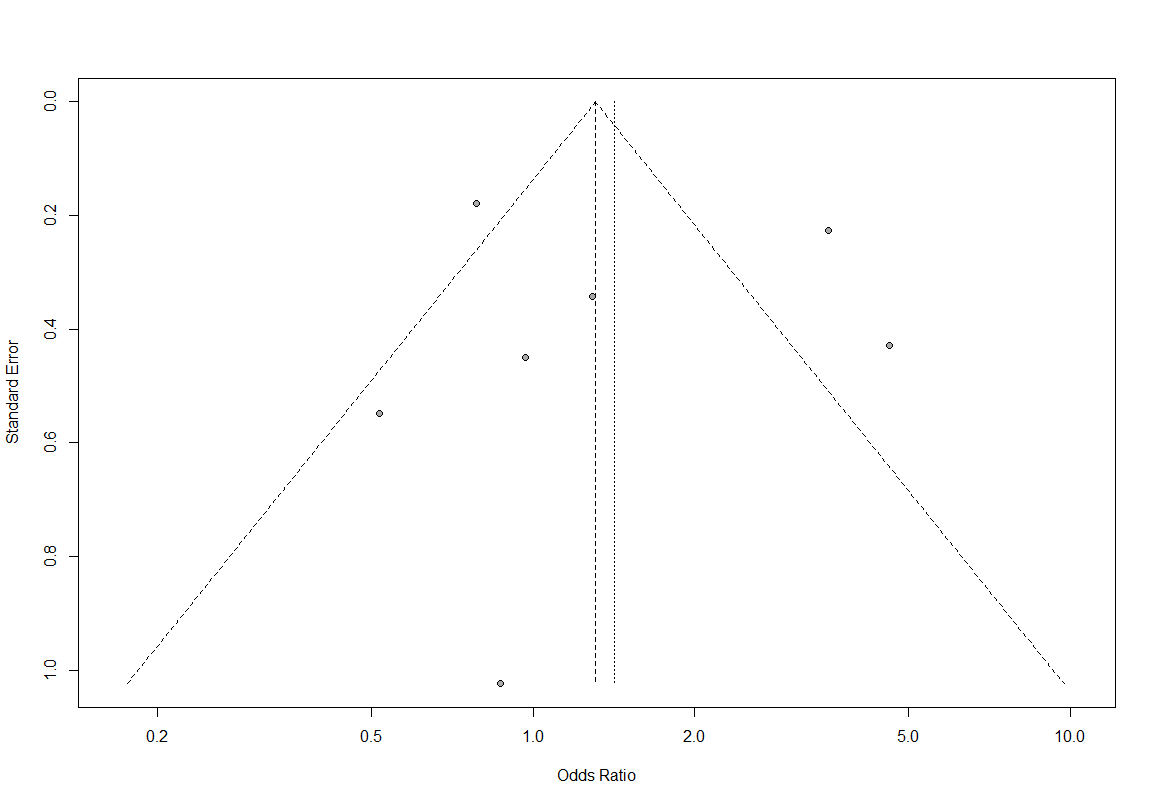


Egger’s test result: t = 0.08, df = 5, p-value = 0.9364

Bias estimate: 0.1959 (SE = 2.3352)

Antiplatelet Treatment


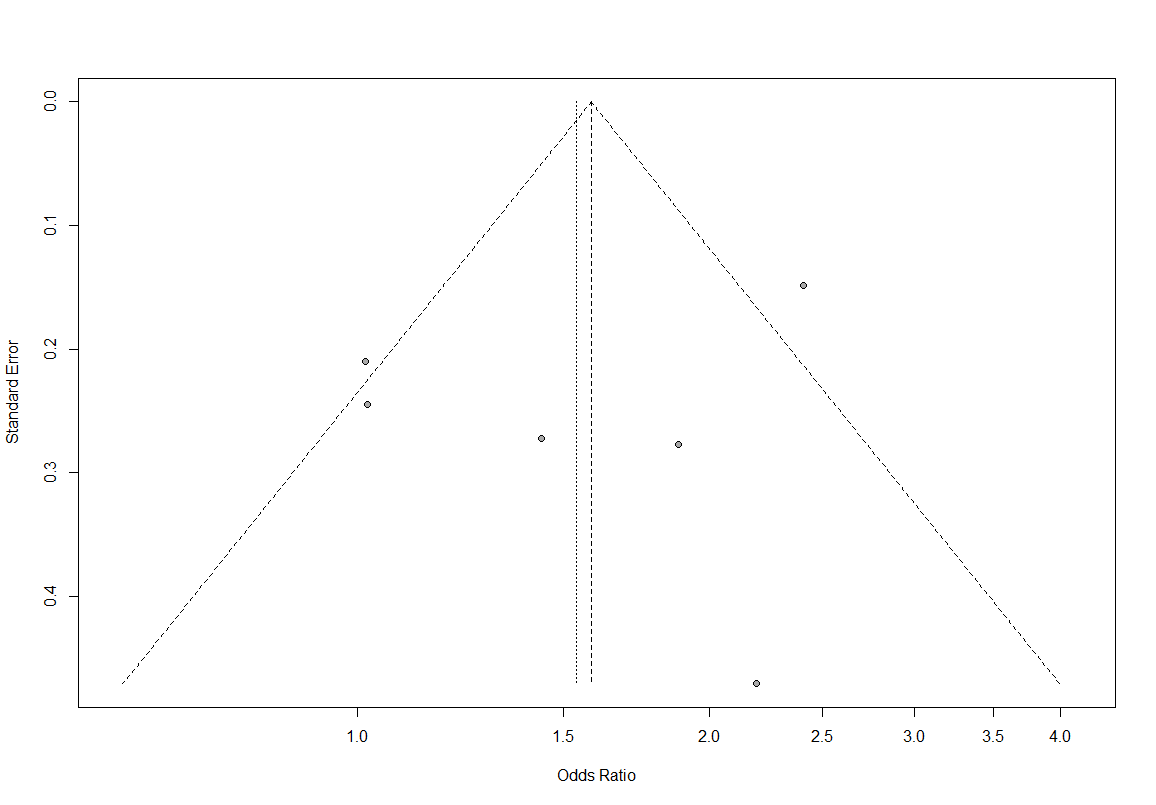


Egger’s test result: t = -0.66, df = 4, p-value = 0.5452

Bias estimate: -1.6386 (SE = 2.4822)

VKA Treatment


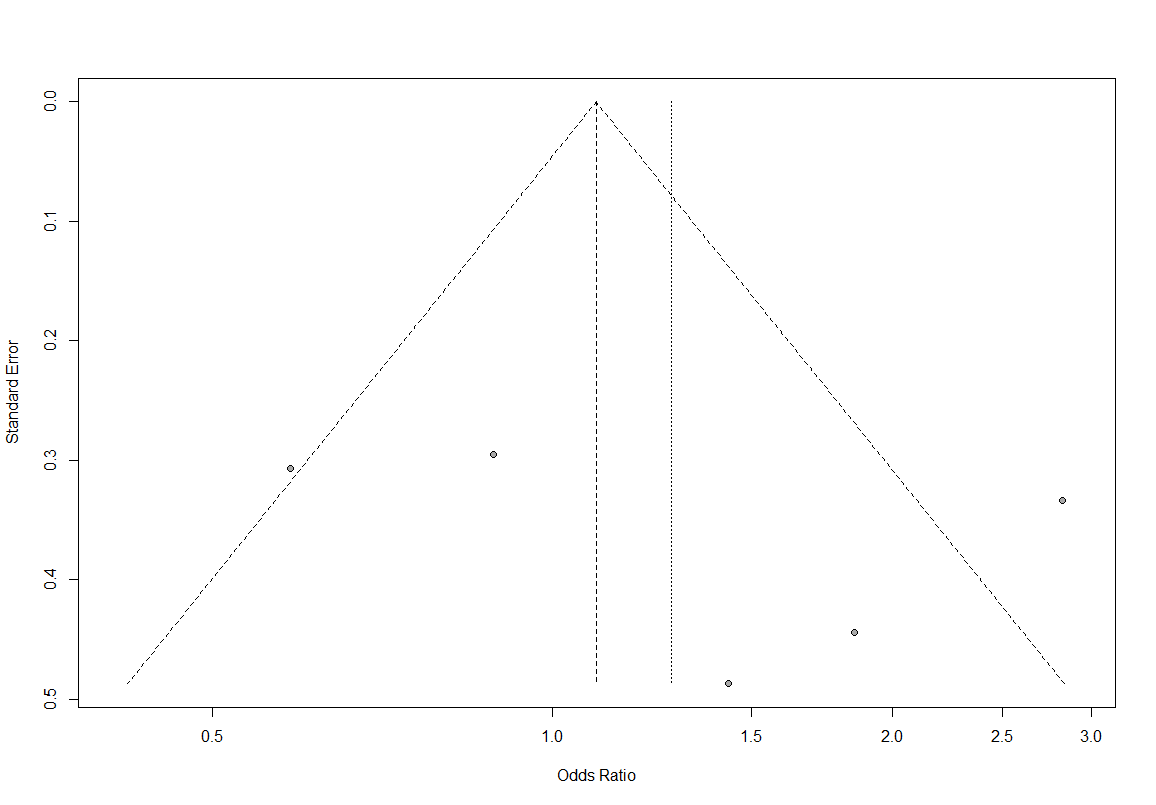


Too few studies for conducting an Egger’s test.

DOAC Treatment


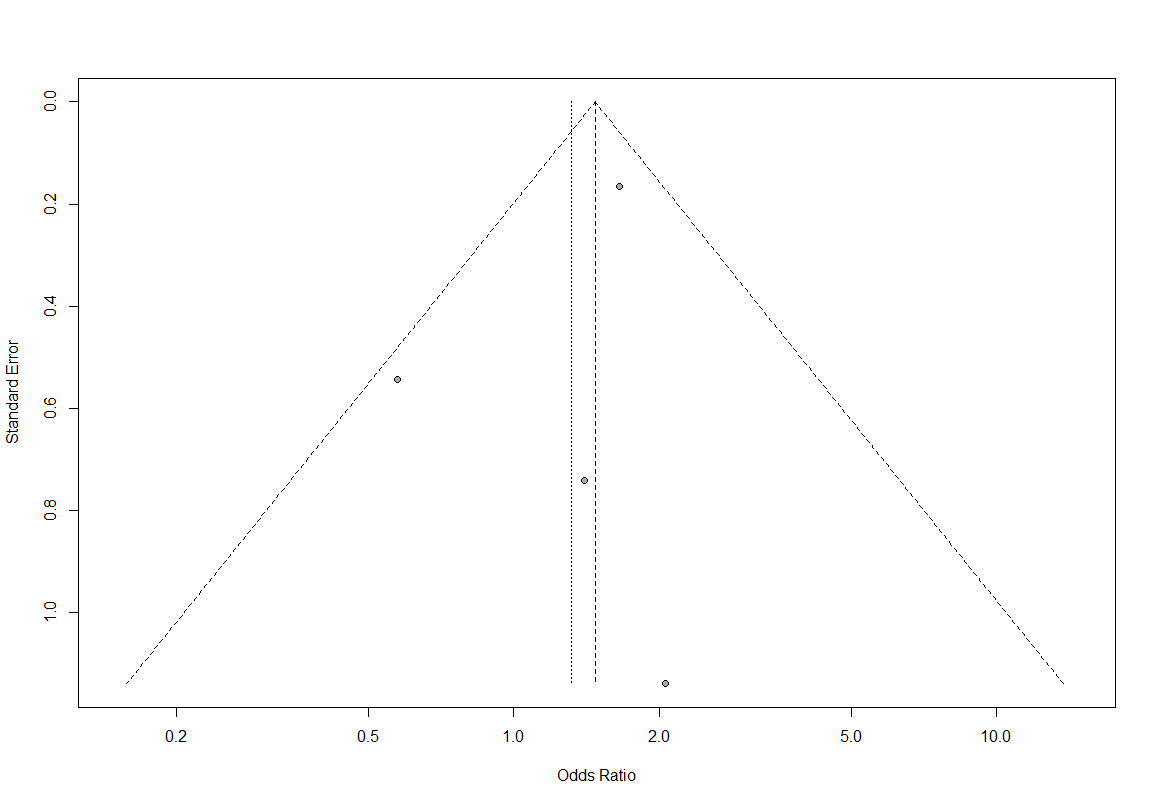


Too few studies for conducting an Egger’s test.

**2. Literature Search Strategy**

Search String:

**((traumatic brain injury) OR TBI OR (brain injury) OR (head injury) OR (head trauma) OR (cerebral trauma) OR (brain trauma) OR (cerebral injury) OR (craniocerebral trauma) OR (cranial trauma) OR (cranial injury) OR concussion) AND (mild OR minor) AND (tomography OR imaging OR CT) AND (hemorrhage OR haemorrhage OR bleed OR blood OR lesion OR insult)**
